# Protective Effects of Ellagic Acid Against Alcoholic Liver Disease in Mice

**DOI:** 10.3389/fnut.2021.744520

**Published:** 2021-09-14

**Authors:** Liang Zhao, Arshad Mehmood, Mohamed Mohamed Soliman, Asra Iftikhar, Maryam Iftikhar, Salama Mostafa Aboelenin, Chengtao Wang

**Affiliations:** ^1^Beijing Advance Innovation Center for Food Nutrition and Human Health, Beijing Engineering and Technology Research Center of Food Additives, Beijing Technology and Business University, Beijing, China; ^2^Clinical Laboratory Sciences Department, Turabah University College, Taif University, Taif, Saudi Arabia; ^3^Department of Pharmacy, Faculty of Pharmaceutical Sciences, The University of Faisalabad, Faisalabad, Pakistan; ^4^Biology Department, Turabah University College, Taif University, Taif, Saudi Arabia

**Keywords:** alcoholic liver disease, polyphenolic compound, ellagic acid, inflammation, gut microbiota

## Abstract

Ellagic acid, a natural polyphenolic compound commonly present in vegetables, fruits, nuts, and other edible plants, exerts many pharmacological activities. The present project was designed to explore the hepatoprotective effect of ellagic acid against alcohol-induced liver disease (ALD) and the correlation among alcohol, oxidative stress, inflammation, and gut microbiota. Fifty percent (v/v) alcohol (10 mL/kg bw daily) was orally administrated for 4 weeks in mice along with ellagic acid (50 and 100 mg/kg bw). Alcohol administration significantly (*p* < 0.05) increased the activities of alanine aminotransferase and serum aspartate aminotransferase, levels of triglyceride, low density lipoprotein, free fatty acid, and total cholesterol, and decreased contents of the high-density lipoprotein in model group compared with the control group, which were further improved by ellagic acid (50 or 100 mg/kg bw). Furthermore, daily supplementation of ellagic acid alleviated hepatic antioxidant activities (glutathione peroxidase, catalase, malondialdehyde, superoxide dismutase, and glutathione), proinflammatory cytokines levels (IL-6, IL-1β, and TNF-α), genes expressions (*Tlr4, Myd88, Cd14, Cox2, Nos2*, and *Nf*κ*b1*), and histopathological features in alcohol-induced liver injured mice. Additionally, results also revealed that ellagic acid supplementation improved alcohol-induced gut microbiota dysbiosis. In conclusion, ellagic acid mitigated oxidative stress, inflammatory response, steatosis, and gut microbiota dysbiosis in ALD mice. Our results suggested that ellagic acid could be applied as an ideal dietary therapy against ALD.

## Introduction

Alcohol-induced liver disease (ALD) is a leading source of morbidity and mortality in Europe, the United States, and China. ALD is a common liver disease and around 300 million people are affected by hepatitis B virus, non-alcoholic fatty liver disease, and ALD in China (1, 2). ALD is characterized by changes in hepatic morphological features such as alcoholic steatohepatitis (ASH), hepatitis, and even cirrhosis (3). The oxidative stress, steatosis, gut microbiota disorders, and inflammatory responses are currently proposed as the main causes of ALD (2, 4–6).

Emerging evidence suggests that consumption of alcohol caused the alternation of gut microbiota composition, known as gut dysbiosis, which plays a significant function in the progression of ASH (6–8). Briefly, chronic alcohol exposure leads to the overgrowth of intestinal bacterial, changes in gut microbiota composition, as well as the systemic elevation of inflammation, endotoxin [lipopolysaccharide (LPS)], and hepatic steatosis (9, 10). Particularly, dysbiosis of the gut microbiota is also found to be linked positively to alcohol-induced hepatic cirrhosis (11, 12). Moreover, results also revealed that relative abundance of *Actinobacteria* and *Firmicutes* markedly increased and *Bacteroidetes* decreased along with the inflammation and hepatic steatosis in alcohol-induced mice (13). Gut dysbiosis increases intestinal permeability, which in turn leads to LPS and gut-derived bacteria entering the liver (14, 15). The circulating endotoxin levels may initiate hepatic inflammation *via* multiple signaling pathways such as Toll-like receptor (TLR) 4, myeloid differentiation primary response gene 88 (MyD88), nuclear factor kappa-B (NF-κB), and others (16, 17). Therefore, gut dysbiosis has a significant impact on ALD, and treating ALD by focusing on the intestinal microbiota is an important clinical approach.

The available options for the treatment of ALD are limited, and most possess some adverse side effects. Therefore, there is an urgent need for alternative safe and inexpensive treatment strategies for coping with ALD. The use of natural products and nutritional agents attracts attention for the treatment of ALD due to their broad spectrum of antiinflammatory and antioxidant properties (5). Polyphenolic compounds have been reported to exert beneficial effects in the prevention and treatment of ALD (18–22). Ellagic acid is a natural polyphenol that belongs to the tannic acid group and is abundantly present in many medicinal plants, fruits, fruits peel of berries and nuts, possessing antiinflammatory, antiapoptotic and antioxidant effects (23–26). Previously studies confirmed that ellagic acid can protect the liver from aceclofenac (27), thioacetamide (28), valproic acid (29), d-galactosamine (30), carbon-tetrachloride (31), and ethanol (32). Previously, an *in vitro* study by Sohn et al. (32) also revealed that ellagic acid attenuated ethanol-induced liver toxicity in HepG2 cells *via* antioxidant and antiinflammatory mechanism. Notably, few studies were conducted before the protective effects of ellagic acid on ALD were understood. Therefore, the present study was designed to investigate the intervention effect of ellagic acid against ALD in mice induced by ethanol, and to further explore the possible mechanism.

## Materials and Methods

### Chemicals and Reagents

Ellagic acid (98%) was purchased from Shanghai Yuanye Biotechnology Co., Ltd. (Nanjing, China). Formalin, sodium carboxymethyl cellulose (CMC-Na), and ethanol were procured from Shanghai Macklin Biochemical Co., Ltd (Shanghai, China). Alanine aminotransferase (ALT), aspartate aminotransferase (AST), total cholesterol (TC), triglycerides (TG), high-density lipoprotein (HDL), low-density lipoprotein (LDL), free fatty acid (FFA), and gamma glutamyl transferase (γGT) kits were procured from Nanjing Jiancheng Bioengineering Institute (Nanjing, China). The malondialdehyde (MDA), superoxide dismutase (SOD), catalase (CAT), glutathione (GSH) and glutathione peroxidase (GSH-Px) kits were procured from Nanjing Jiancheng Bioengineering Institute (Nanjing, China). The enzyme-linked immunosorbent assay (ELISA) kits (IL-6, IL-1β, and TNF-α) were purchased from Beijing Sinouk institute of biological technology (Beijing, China). TRIzol reagent and FastQuant RT kit were purchased from Tiangen Biotech Co., Ltd. (Beijing, China). All other chemicals used in the present study were analytical grade.

### Animals

Forty male ICR mice, aged 7–9 weeks and weighing 22–24 g, were procured from Beijing Vital River Laboratory Animal Technology Co., Ltd. (Beijing, China) (Certificate SCXK (Beijing) 2012-0001). The mice were kept in a well-ventilated animal room for a 1-week acclimation period at 25°C, humidity (60–80%), under 12 h light/12 h dark cycle. The animal experiment project was approved by the Ethics Committee of the Beijing Key Laboratory of Functional Food from Plant Resources (Permit number: A330-19). All animal experimental procedures in this research also followed the National Institutes of Health's guidelines for the treatment and use of laboratory animals.

### Design of Animal Experiment

After 1-week acclimation period, four groups of mice were formed (*n* = 10 in each group); control group (C), model group (M), low-dose of ellagic acid group (EL), and high-dose of ellagic acid group (EH); and were fed with normal pellet diet *ad libitum* and water. The C group was given CMC-Na (0.5%) *via* oral way whereas M group was administered with 50% (v/v) alcohol (10 mL/kg bw daily) after 1 h of 0.5% CMC-Na by oral route for 4 weeks. The mice in EL and EH were administered orally with low (50 mg/kg bw) or high (100 mg/kg bw) dose of ellagic acid (suspended in 0.5% CMC-Na) after 1 h of receiving 50% alcohol for 4 weeks. Every 3 days, the mice were weighted, and the amount of gastric infusion received was adjusted based on the weight. The total animal experiment lasted for 4 weeks. After 4 weeks, all mice were fasted for 12 h before being weighed and killed. Using a capillary tube, blood samples were taken from each mouse retroorbital venous plexus. Liver, kidney, and spleen tissues were dissected out, rinsed, washed with ice-cold PBS, and weighed to measured organ index according to the following formula:

Organ index (%) = organ weight/final body weight × 100%

Following that, one section of the liver tissues was immersed in a 10% formaldehyde solution for histopathological examination whereas the remaining sections were kept for biochemical study, ELISA determination, and qPCR measurement.

### Biochemical Analysis of Liver Tissues and Serum

The blood samples were clotted at 4°C for 6 h and then centrifugated at 4,000 g for 15 min to obtain the serum. After that, the serum was further subjected for the determination of various biochemical parameters such as ALT, AST, TC, TG, HDL, LDL, FFA, and γGT by using commercially available kits according to the instruction manuals.

Liver tissue homogenates were prepared, and the lipids were separated according to the previously described method (13). Bicinchoninic acid protein assay kit was used to assess the total protein concentrations in the liver homogenate. The hepatic lipid profiles such as TG, TC, and FFA were measured by using the same protocol described for serum FFA, TC, and TG levels. The antioxidant activities such as MDA, SOD, CAT, GSH, and GSH-Px levels in liver tissue homogenates were examined by using commercially available kits following the protocol of the manufacturer.

### Determination of Hepatic Proinflammatory Cytokines

The IL-6, IL-1β, and TNF-α (proinflammatory cytokines) concentrations in the liver were determined by using commercial ELISA kit, following the instruction manual.

### Histological Investigation of Liver

Samples of liver were separated from each mouse and fixed in formalin solution (10%) for 24 h, and after that dehydrated using graded alcohol and xylene, and implanted in paraffin. Paraffin implanted segments were further cut into the thickness of 5 μm, stained with Masson's trichrome (MAS) and hematoxylin and eosin (H&E) for histological investigation. The degree of histological damage to the liver sections was further determined by using a light microscope (BA-9000L, Osaka, Japan).

### Real-Time PCR Analysis

The total RNA was isolated from hepatic tissue by using TRIzol reagent according to the method reported by Mehmood et al. (33). RNA was reverse transcribed to cDNA using a FastQuant RT kit, and mRNA expression was quantified using real time polymerase chain reaction (qRT-PCR). The details of primers used in this study are presented in Table 1. To normalize mRNA expression, the expression of the housekeeping gene, glyceraldehyde-3-phosphate dehydrogenase (GAPDH), was measured.

**Table 1:** List of primer sequences of genes used in this study.

**Gene**		**Primer sequence (5′-3′)**
*Gapdh*	Forward:	CCTTCATTGACCTCAACTACATGGT
	Reverse:	TCATTGTCATACCAGGAAATGAGCT
*Cd14*	Forward:	GGAAGCCAGAGAACACCATC
	Reverse:	CCAGAAGCAACAGCAACAAG
*Myd88*	Forward:	AGAACAGACAGACTATCGGCT
	Reverse:	CGGCGACACCTTTTCTCAAT
*Nfkb1*	Forward:	AGACAAGGAGCAGGACAT
	Reverse:	CCAGCAACATCTTCACATC
*Cox2*	Forward:	GGAGAGACTATCAAGATAGTGATC
	Reverse:	ATGGTCAGTAGACTTTTACAGCTC
*Nos2*	Forward:	GTGGTGACAAGCACATTTGG
	Reverse:	GGCTGGACTTTTCACTCTGC

### Gut Microbiota Analysis

Colonic contents in mice were collected in individual sterile cryotube and stored in liquid nitrogen (34). The total DNA was extracted *via* the EZNA soil DNA extraction kit (Omega Bio-tek, Norcross, USA) according to instruction manuals. The V3–V4 region of the bacterial 16S ribosomal RNA gene was subjected to PCR amplification using primers 338F (5′-ACTCCTACGGGAGGCAGCAG-3′) and 806R (5′ -GGACTACHVGGGTWTCTAAT-3′).

The Illumina MiSeq sequencing technology along with multivariate statistical methods were conducted to detect the diversity of the V3-V4 region of the bacterial 16S rRNA gene. Related library construction and the Miseq high-throughput sequencing process were performed by Majorbio (Shanghai, China). Operational taxonomic units (OTUs) were clustered at 97% similarity, and Venn diagram, analyses of community composition, and Spearsman correlation analysis were performed.

### Statistical Analysis

The data of the animal experiment was evaluated by using SPPSS version 22.0 (SPSS Inc., Chicago, IL, USA) and Graph Pad Prism version 8 (La Jolla, CA, USA). One-way analysis of variance (ANOVA) was used to compare variations between groups, followed by Duncan's multiple range test. Differences between groups were found statistically significant at *p* < 0.01 or *p* < 0.05 and the data was expressed as mean ± SD.

## Results

### Effect of Ellagic Acid on Food Intake, Body Weight, and Organ Index in ALD Mice

The food intake by different groups of mice was measured in g/day. The normal group (C) utilized a healthy amount of food throughout the period of the experiment (Table 2). Compared with the normal group, food utilization in the model group (M) after alcohol administration was prominently decreased (*p* < 0.05), resulting in significant loss of body weight and activities (*p* < 0.05). However, the feed intake and weight loss were significantly improved in the two ellagic acid groups (EL and EH, *p* < 0.05), which was close to the C group. As shown in Table 2, the liver index of the M group was prominently raised compared with mice in the C group (*p* < 0.05). However, the administration of different doses of ellagic acid (EL and EH) attenuated the liver swelling compared with the M group. In addition, there were no significant changes in organ indices of kidney and spleen among mice in C, M, EL, and EH groups (*p* > 0.05).

**Table 2:** Effect of ellagic acid on food intake, body weight, and organ index in mice.

**Variable**	**C**	**M**	**EL**	**EH**
Food intake, g/day	4.65 ± 0.35^a^	3.22 ± 0.52^b^	4.18 ± 0.57^a^	4.20 ± 0.55^a^
Initial body weight, g	25.51 ± 0.97^a^	25.39 ± 0.88^a^	25.91 ± 0.81^a^	25.82 ± 0.83^a^
Final body weight, g	33.01 ± 2.84^a^	26.63 ± 1.34^b^	29.90 ± 2.11^a^	30.13 ± 2.71^a^
Liver index, %	3.85 ± 0.30^a^	4.65 ± 0.34^b^	4.00 ± 0.20^a^	3.98 ± 0.31^a^
Kidney index, %	1.45 ± 0.11^a^	1.67 ± 0.30^a^	1.60 ± 0.30^a^	1.57 ± 0.30^a^
Spleen index, %	0.23 ± 0.04^a^	0.28 ± 0.02^a^	0.25 ± 0.03^a^	0.26 ± 0.05^a^

*Values represent the mean ± SD (n ≥ 6). Labeled means without a common letter difference. p < 0.05 by one-way ANOVA followed by Duncan's test. C, normal group; M, model group; EL, low dose of ellagic acid group; EH, high dose of ellagic acid*.

### Effect of Ellagic Acid on Serum Biomarkers and Lipids Profile in ALD Mice

**Figure 1:**
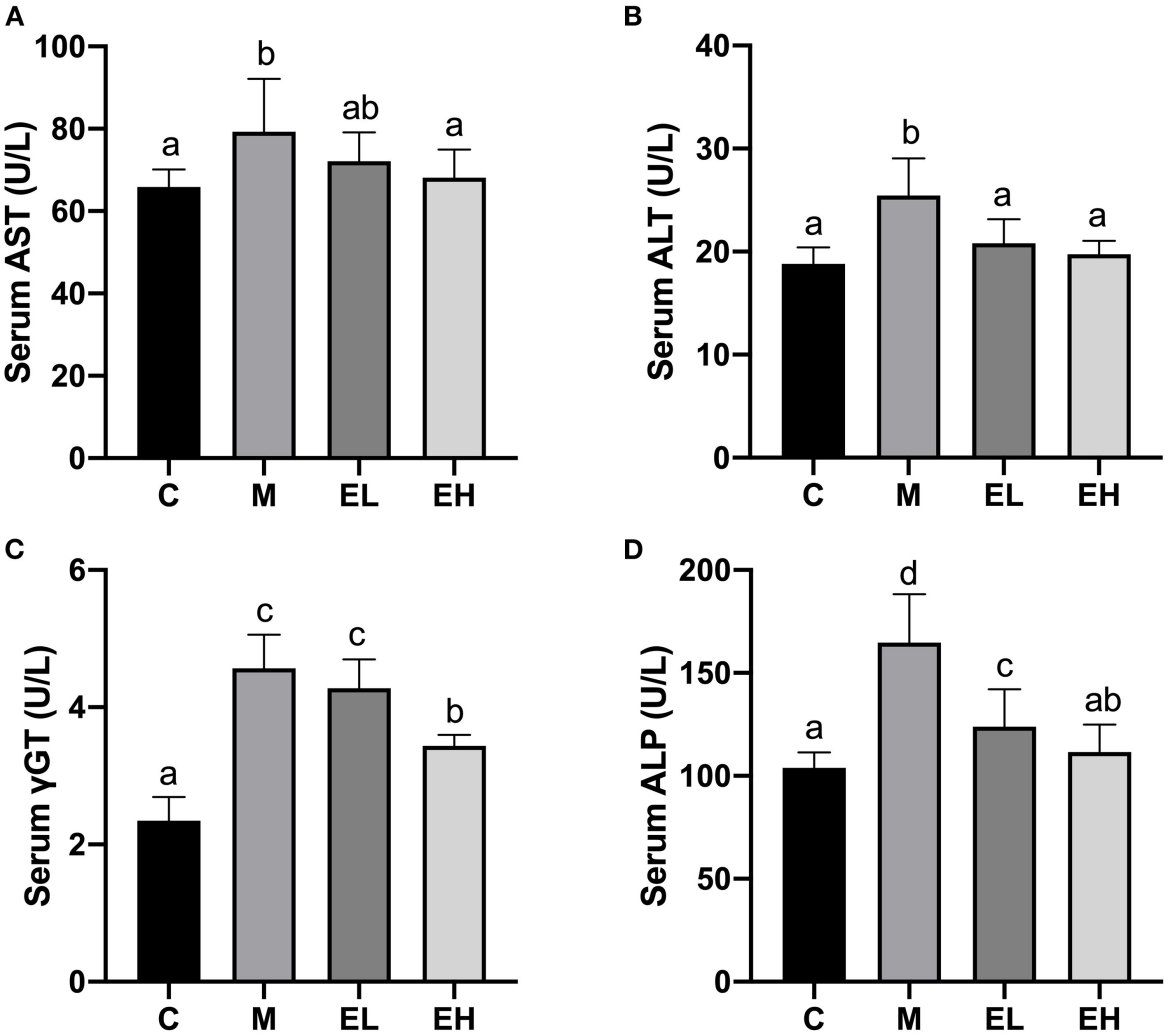
Effect of ellagic acid on serum biochemical makers in mice. **(A)** AST; **(B)** ALT; **(C)** γGT; **(D)** ALP. Values represent the mean ± SD (*n* ≥ 6). Labeled means without a common letter difference. *p* < 0.05 by one-way ANOVA followed by Duncan's test. C, normal group; M, model group; EL, low dose of ellagic acid group; EH, high dose of ellagic acid group.

As displayed in Figures 1A–D, a significant elevation (*p* < 0.05) in the serum AST (Figure 1A), ALT (Figure 1B), γGT (Figure 1C), and ALP (Figure 1D) activities occurred in the M group when compared with the C group. The treatment with ellagic acid (EL and EH) markedly (*p* < 0.05) downregulated the activities of the enzymes; AST, ALT, γGT, and ALP; when compared with the M group.

**Figure 2:**
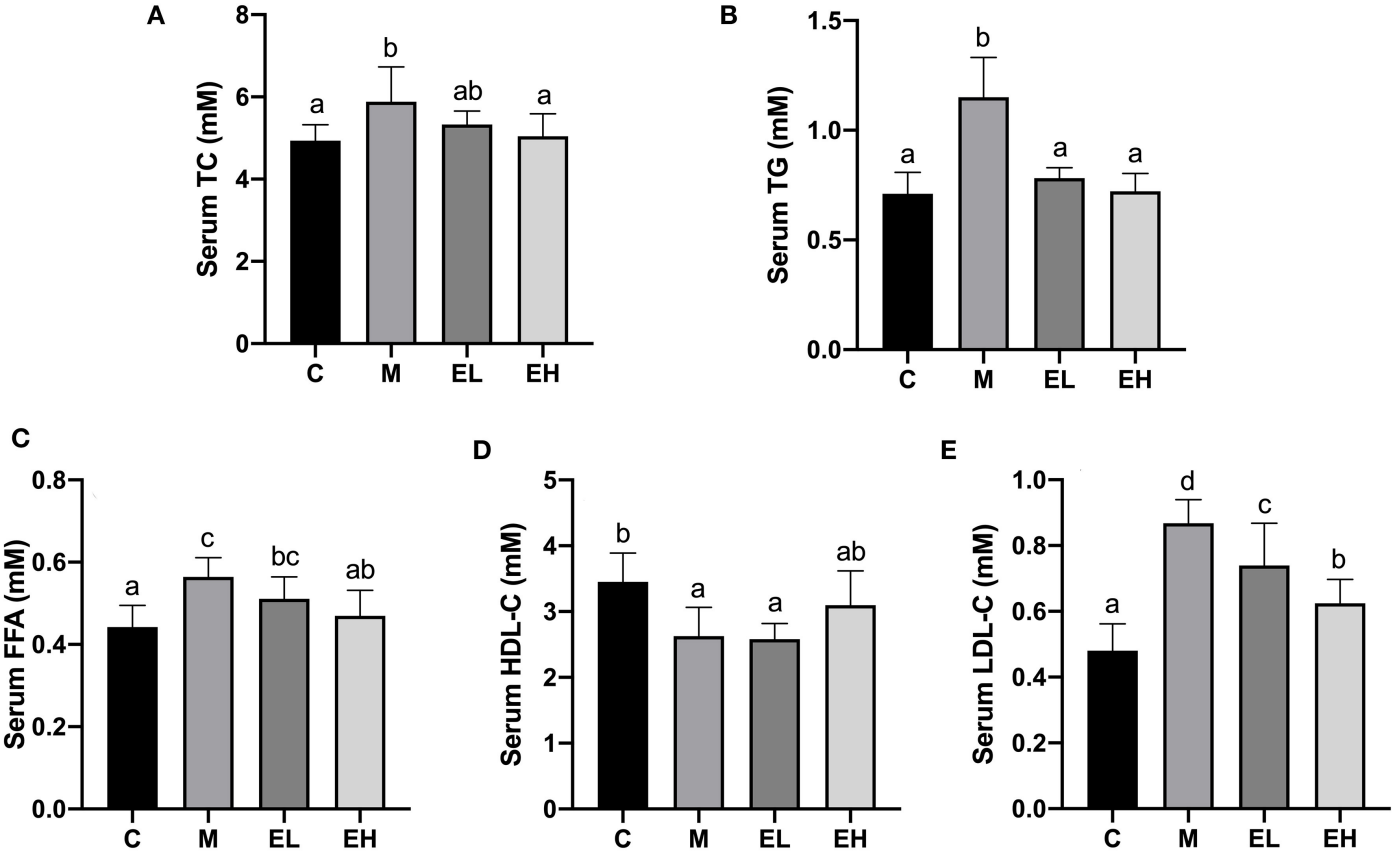
Effect of ellagic acid on serum lipid profile in mice. **(A)** TC; **(B)** TG; **(C)** FFA; **(D)** HDL-C; **(E)** LDL-C. Values represent the mean ± SD (*n* ≥ 6). Labeled means without a common letter difference. *p* < 0.05 by one-way ANOVA followed by Duncan's test. C, normal group; M, model group; EL, low dose of ellagic acid group; EH, high dose of ellagic acid group.

The results regarding serum lipid profiles such as TG, TC, HDL-C, LDL-C, and FFA are presented in Figures 2A–E. Compared with the C group, a significant rise in the serum TC, TG, LDL-C, and FFA levels and a downregulation of HDL-C level were observed in the M group (*p* < 0.05). Interestingly, the serum TG, TC, HDL-C, LDL-C, and FFA levels were significantly downregulated (*p* < 0.05), and HDL-C level was upregulated by ellagic acid treatment (EL and EH) compared with the M group.

### Effect of Ellagic Acid on Hepatic Lipids Profile of Alcohol-Induced ALD Mice

**Figure 3:**
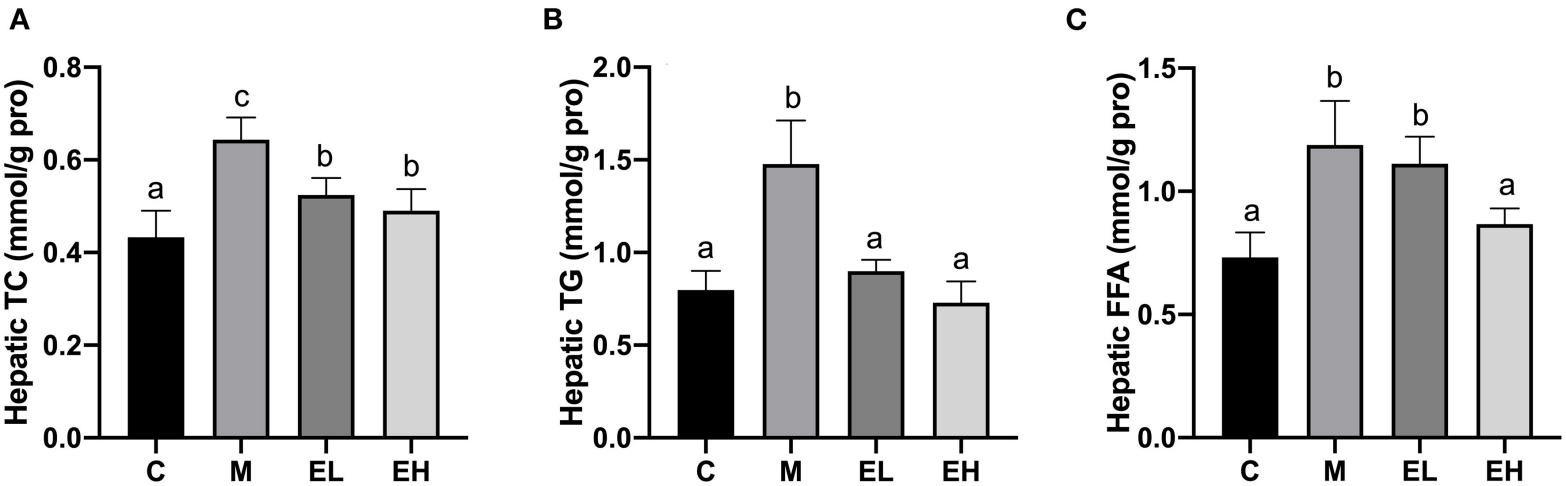
Effect of ellagic acid on hepatic lipid profile in mice. **(A)** Hepatic TC; **(B)** Hepatic TG; **(C)** Hepatic FFA. Values represent the mean ± SD (*n* ≥ 6). Labeled means without a common letter difference. *p* < 0.05 by one-way ANOVA followed by Duncan's test. C, normal group; M, model group; EL, low dose of ellagic acid group; EH, high dose of ellagic acid group.

As shown in the Figures 3A–C, hepatic lipid indicators such as TC (Figure 3A), TG (Figure 3B), and FFA (Figure 3C) were markedly upregulated in M group compared with the C group (*p* < 0.05). Ellagic acid treatment (EL and EH) induced a significant decline in the hepatic lipid indicators, e.g., TC, TG, and FFA (*p* < 0.05). However, EL failed to restore FFA level compared to the M group.

### Effect of Ellagic Acid on Hepatic Oxidative Stress Parameters in ALD Mice

**Figure 4:**
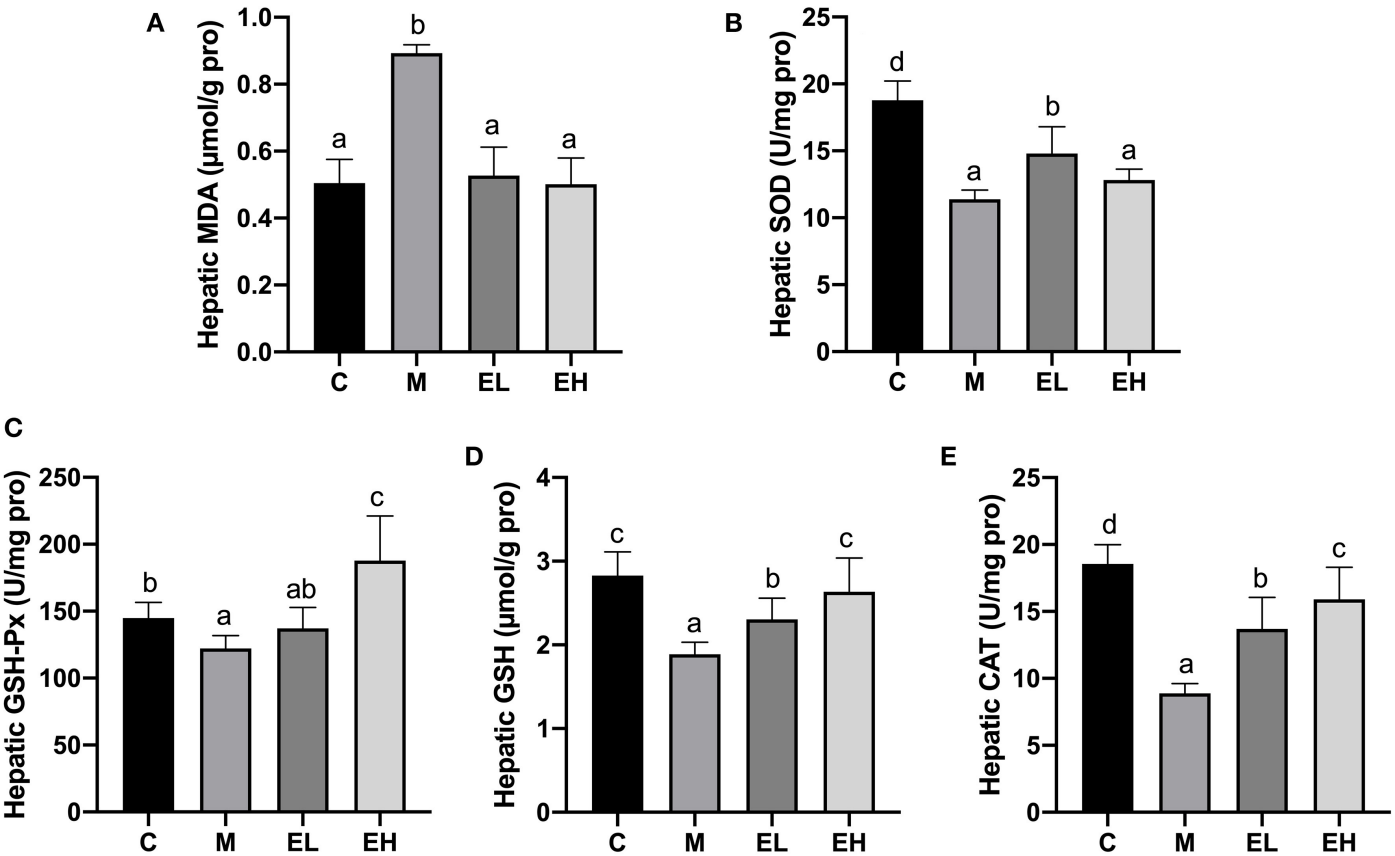
Effect of ellagic acid on hepatic oxidative stress parameters in mice. **(A)** MDA; **(B)** SOD; **(C)** GSH-Px; **(D)** GSH; **(E)** CAT. Values represent the mean ± SD (*n* ≥ 6). Labeled means without a common letter difference. *p* < 0.05 by one-way ANOVA followed by Duncan's test. C, normal group; M, model group; EL, low dose of ellagic acid group; EH, high dose of ellagic acid group.

One of the major pathological events during the development of alcoholic fatty liver disease is oxidative stress since it connects lipid metabolism dysfunction to downstream inflammation and apoptosis (2, 4). Antioxidant enzymes, e.g., GSH-Px, CAT, MDA, SOD, and GSH, play significant roles in protecting alcohol-induced hepatic damage and oxidative stress. Figures 4A–E showed the effect of ellagic acid on hepatic antioxidant enzymes (GSH-Px, CAT, MDA, SOD, and GSH). Hepatic MDA level significantly elevated in the M group compared with C group (*p* < 0.05). The ellagic acid-treated groups (EL and EH) markedly decreased the MDA content compared with the M group (*p* < 0.05). In addition, hepatic GSH-Px, CAT, SOD, and GSH levels were significantly decreased in M group compared with C group (*p* < 0.05), which was restored by ellagic acid administration (EL and EH groups). The results clearly indicated that ellagic acid showed strong potential to alleviate hepatic damage in alcohol-induced mice *via* downregulating MDA content and increasing the antioxidant enzymes (GSH-Px, CAT, MDA, SOD, and GSH).

### Effect of Ellagic Acid on the Hepatic Histopathological Features of Alcohol-Induced ALD Mice

**Figure 5:**
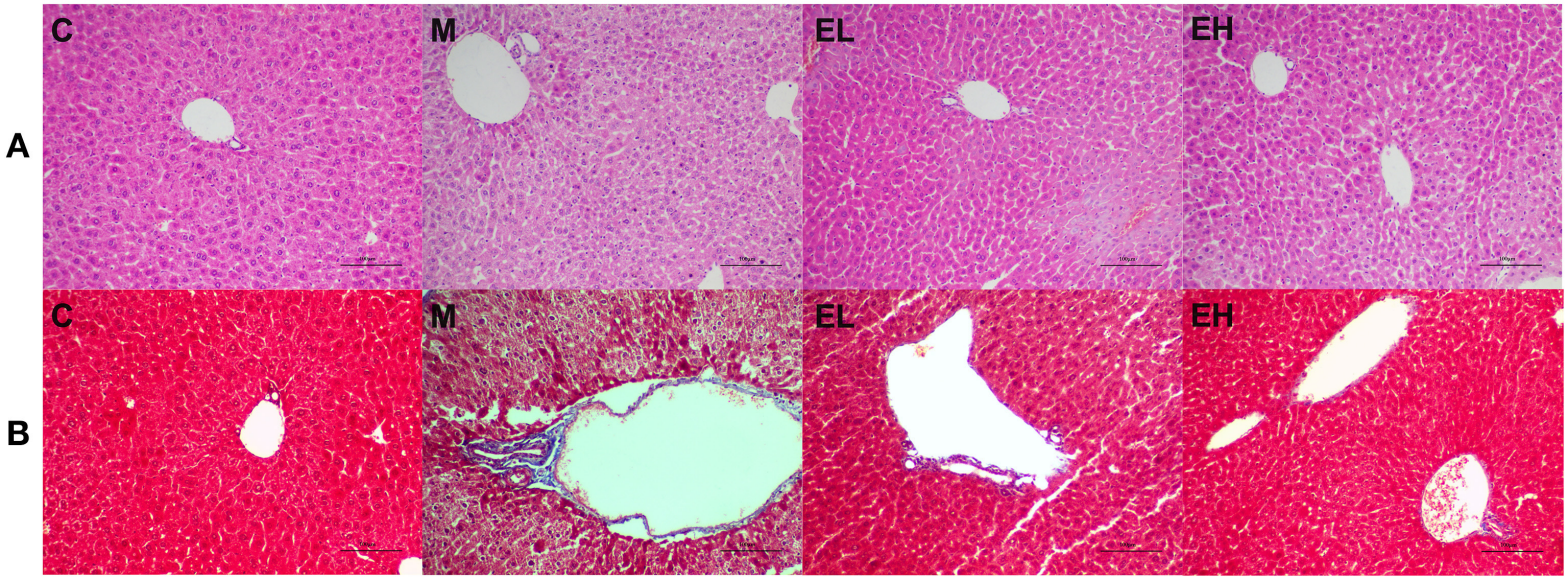
Effect of ellagic acid on hepatic histopathology feature of alcohol induced ALD mice. **(A)** H&E staining; **(B)** MAS staining. C, normal group; M, model group; EL, low dose of ellagic acid group; EH, high dose of ellagic acid group.

To determine the protective effect of ellagic acid against ALD, we investigated the histopathological changes in the hepatic tissue by using H&E and MAS staining assay. As presented in the Figure 5, the structure of liver lobule was complete and clear with no evident inflammatory cell infiltration, and hepatocytes were in an ordered arrangement with centrally and round located nuclei in C group mice. However, after alcohol exposure (M group), a significant inflammatory cell infiltration, fibrosis, and enlargement of hepatocytes around the central vein was observed. The ellagic acid-treated groups (EL and EH) exhibited less edema, fibrosis, and inflammatory cell infiltration compared with M group.

### Effect of Ellagic Acid on Hepatic Proinflammatory Cytokines Levels in ALD Mice

**Figure 6:**
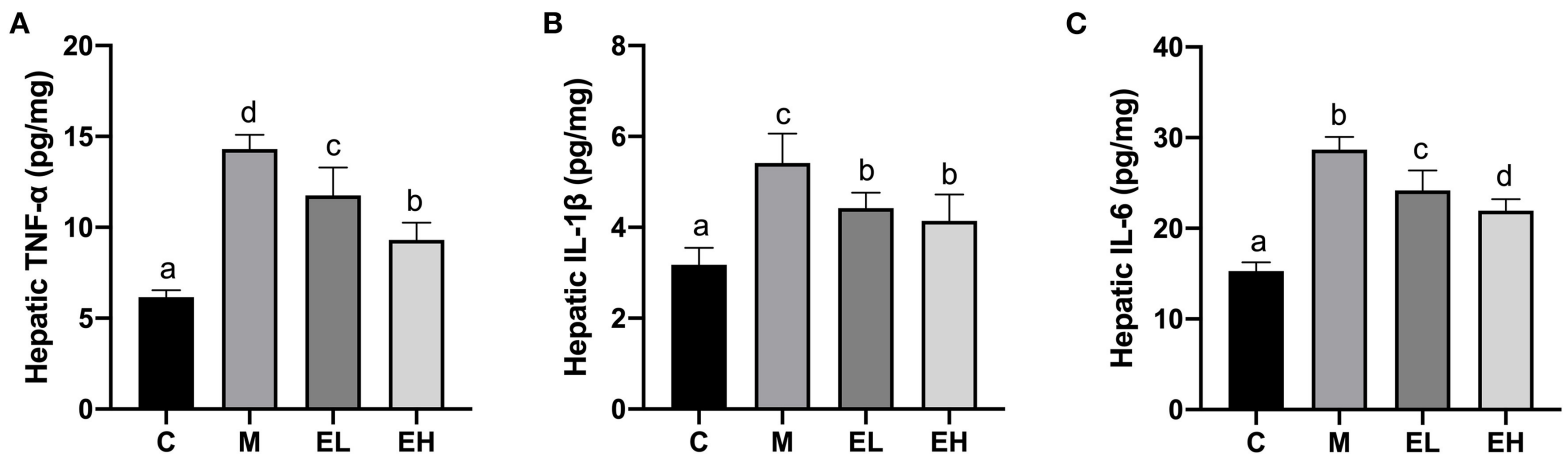
Effect of ellagic acid on hepatic pro-inflammatory cytokines levels in mice. **(A)** TNF-α; **(B)** IL-1β; **(C)** IL-6. Values represent the mean ± SD (*n* ≥ 6). Labeled means without a common letter difference. *p* < 0.05 by one-way ANOVA followed by Duncan's test. C, normal group; M, model group; EL, low dose of ellagic acid group; EH, high dose of ellagic acid group.

The results regarding proinflammatory cytokines such as IL-6, IL-1β, and TNF-α in the hepatic tissues are presented in the Figures 6A–C. A significant (*p* < 0.05) elevation in the hepatic IL-6, IL-1β, and TNF-α were observed in M group mice compared with C group. Interestingly, treatment of ellagic acid (EL and EH) was able to markedly decrease the levels of TNF-α, IL-1β, and IL-6 compared with the M group (*p* < 0.05). These results suggested that ellagic acid treatment was an effective approach to attenuate alcohol-induced hepatic inflammation.

### Effect of Ellagic Acid on the Hepatic Inflammation Associated Genes Expression of ALD Mice

**Figure 7:**
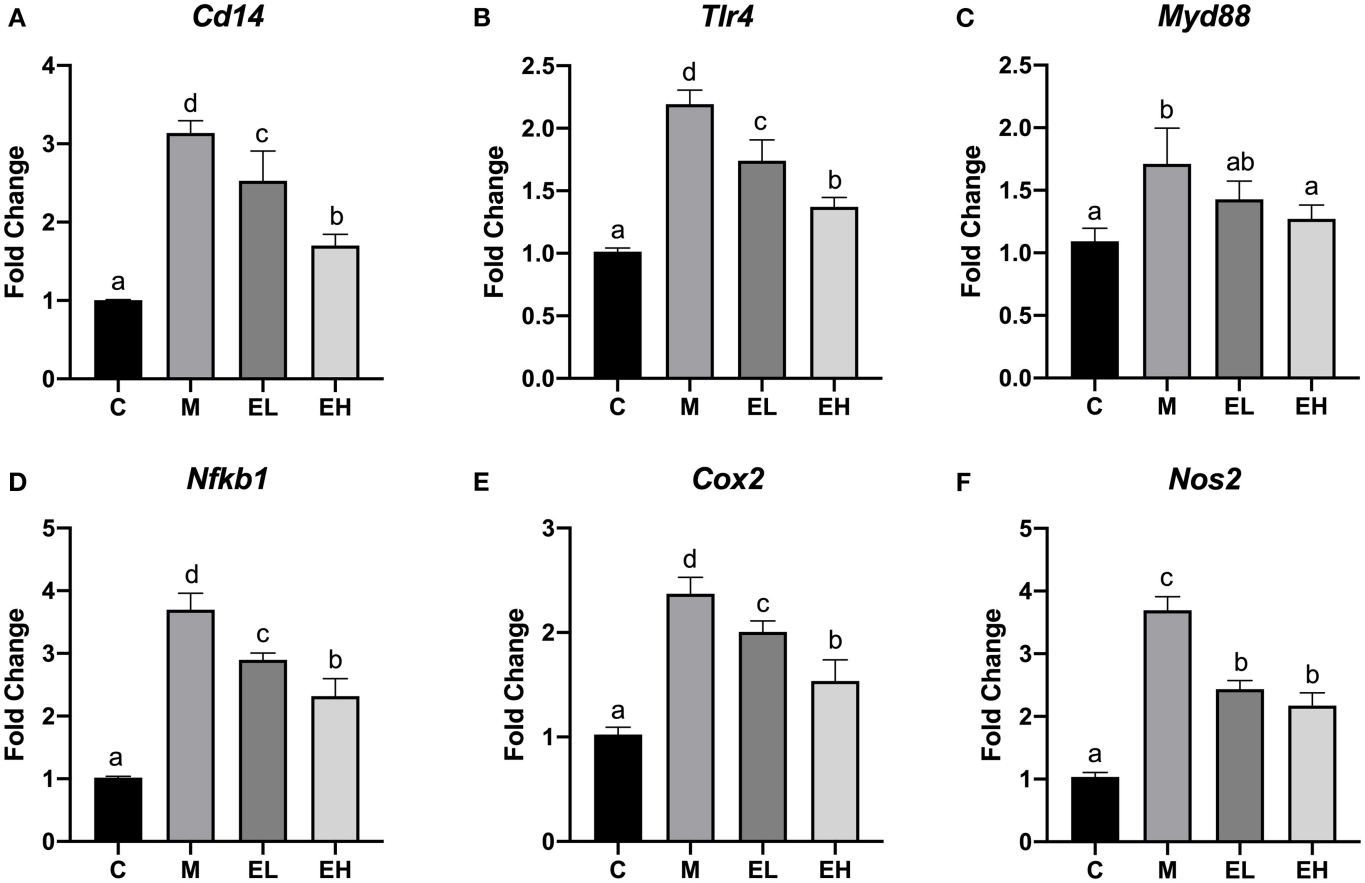
Effect of ellagic acid on the hepatic inflammation associated genes expression in mice. **(A)** *Cd14*; **(B)** *Tlr4*; **(C)** *Myd88*; **(D)** *Nfkb1*; **(E)** *Cox2*; **(F)** *Nos2*. Values represent the mean ± SD (*n* ≥ 3). Labeled means without a common letter difference. *p* < 0.05 by one-way ANOVA followed by Duncan's test. C, normal group; M, model group; EL, low dose of ellagic acid group; EH, high dose of ellagic acid group.

According to the results, hepatic mRNA expression of TLR4, a trigger of inflammation in ALD was significantly increased in M group mice (*p* < 0.05), which further caused the upregulation of MyD88 and downstream genes compared with the C group (Figures 7A–F). However, it has been observed that EL and EH significantly reduced the expression of TLR4 (*Tlr4*) and downstream MyD88 genes (*Myd88*) in a dose-dependent manner resulting in reduced inflammatory changes (*p* < 0.05). Expression of inflammatory mediators (NF-κB and COX-2) and a cluster of differentiation (CD14) was significantly higher in the liver of M group of mice (*p* < 0.05). However, treatment with ellagic acid considerably reversed the inflammatory necrosis by downregulating the NF-κB (*Nfkb1*), COX-2 (*Cox2*), and CD14 (*Cd14*) genes expression (*p* < 0.05). Furthermore, a high dose of ellagic acid (100 mg/kg bw) was more effective in inhibiting the overexpression of TLR4, COX-2, CD14, and NF-κB genes than the low dose of ellagic acid (50 mg/kg bw, *p* < 0.05). It was also noticed that both doses of ellagic acid (EL and EH) reduced the hepatic inflammation by suppressing the expression of iNOS gene (*Nos2*) compared with the M group.

### Effect of Ellagic Acid on the Intestinal Microbial Composition in ALD Mice

**Figure 8:**
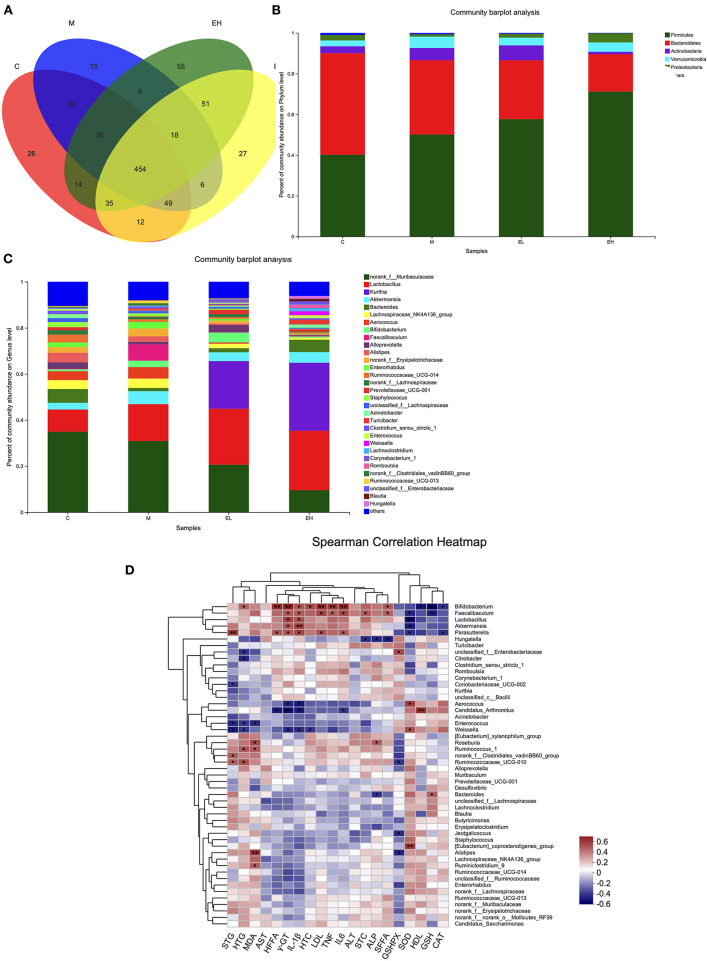
Effect of ellagic acid on the intestinal microbial composition in ALD mice. **(A)** Venn plot; **(B)** Bacterial profile at phylum level; **(C)** Bacterial profile at genus level; **(D)** Heatmap of Spearman correlation analysis of physical parameters and microbiota based on species level. Values represent the mean ± SD (*n* ≥ 5). C, normal group; M, model group; EL, low dose of ellagic acid group; EH, high dose of ellagic acid group.

In the Figure 8A, Venn chart showed the number and overlap of sample OTUs in each and among the group. The results revealed that a total of 454 OTUs were shared by all the samples. At the phylum level (Figure 8B), ethanol administration elevated the relative abundance of *Firmicutes, Verrucomicrobia, Actinobacteria*, and decreased the relative abundance of *Bacteroidetes* and *Proteobacteria* compared with the C group. The administration of ellagic acid markedly modulated gut microbiota to some extent such as decreasing the relative abundance of *Actinobacteria* and *Verrucomicrobia* compared with the model group. At the genus level (Figure 8C), the *norank_f__Muribaculaceae, Lactobacillus, Kurthia, Akkermansia, Bacteroides*, and *Lachnospiraceae_NK4A136_*group were found to be the most prevalent six genera in colonic samples of tested mice. Alcohol administration decreased the relative abundance of *norank_f__Muribaculaceae* and *Bacteroides*, which were modulated to some extent by the treatment of EL and EH. In addition, the relative abundance of *Bacteroides* increased and *Lachnospiraceae_NK4A136_*group decreased in the EH group compared with the M group. The EL also improved the relative abundance of *Bifidobacterium* compared with the M group. Collectively, our results indicated that ellagic acid could significantly modulate gut microbiota composition.

Correlations between the microbiome and several biochemical indicators were tested by Spearman's correlation analysis. As shown in Figure 8D, the relative abundance of *Bifidobacterium* was significantly correlated with hepatic FFA, γGT, LDL, IL-6, and GSH. The relative abundance of *Lactobacillus* was significantly correlated with SOD. The relative abundance of *Akkermansia* was significantly correlated with IL-1β. The relative abundance of *Parasutterella* was significantly correlated with serum TG. The relative abundance of *Candidatus_Arthromitus* was significantly correlated with γGT and HDL. The relative abundance of (*Eubacterium*)_*coprostanoligenes*_group was significantly correlated with SOD. The relative abundance of *Alistipes* was significantly correlated with MDA.

## Discussion

Chronic alcohol intake could lead to ALD such as alcoholic fatty liver (AFL), steatohepatitis, and cirrhosis. The pathogenesis of ALD is very complex and may be associated with oxidative stress (OS) and inflammatory responses. Notably, despite years of ongoing research, the mechanisms of ALD remain obscure. Interestingly, recent studies confirmed that natural products (black rice, aged black garlic, Pinus thunbergii Parl, noni fruit, etc.) and bioactive compounds (apigenin, quercetin, naringenin, (-)-epigallocatechin gallate, genistein, and platycodin D, etc.) could attenuate ALD via multiple pathways (18–20, 22, 35–39). Based on the broad spectrum of antioxidant and antiinflammatory effects of ellagic acid, the present study was designed to evaluate the hepatoprotective effect of ellagic acid against ALD.

Fatty liver is the earliest pathology of ALD, in which hepatocytes contain macrovesicular droplets of TG. It was reported that AFL is a result of fat metabolism imbalance, such as increased TG synthesis along with decreased mitochondrial lipid oxidation (40). Serum AST and ALT are also important biochemical indicators for hepatic cellular injury. Under the healthy condition, ALT and AST are present in hepatocytes. However, in the event of hepatic damage, these enzymes are released from hepatocytes into circulation (41). In the present study, we noticed that the consumption of alcohol markedly raised the serum TG level, and activities of ALT and AST, which clearly indicated the occurrence of hepatic injury caused by alcohol. However, the ellagic acid supplementation (at the dosage of 50 and 100 mg/kg bw) significantly alleviated lipid metabolism disorder and hepatocyte integrity in ALD mice, which is correlated with many previous reports in the same animal model (18, 37–39, 42–44).

Oxidative stress plays an important role in ALD. The consumption of alcohol aggravated OS *via* elevating reactive oxygen species (ROS). In the normal physiological environment, there are many antioxidants present in cells such as SOD, CAT, GSH, vitamin E, etc., which can eliminate harmful free radicals in the body. Therefore, the elimination and production of free radicals should be in dynamic equilibrium. However, excessive alcohol intake can lead to a loss of this dynamic equilibrium, and a higher generation of ROS surpasses the production of abundant antioxidants to remove ROS, causing OS and cell damage. Nrf2 (NF-E2-related factor 2) plays a key role in maintaining and regulating OS response mediated by alcohol, which can inhibit the OS-induced inflammation (3–5). In this study, protective effects of ellagic acid on the hepatic activities or levels of MDA, GSH, CAT, GSH-Px, and SOD were evaluated, and results revealed that ellagic acid markedly improved antioxidant defense status *via* increasing the hepatic GSH, CAT, GSH-Px, and SOD activities and decreasing the MDA content in ALD mice. Girish and Pradhan (31) documented that ellagic acid and curcumin treatment decreased hepatic MDA levels in CCl_4_-induced liver toxicity in mice. In another study, Ding et al. (45) also reported that ellagic acid inhibited ROS release in CCl_4_-induced cirrhotic mice. In addition, Girish et al. (46) observed that ellagic acid reduced the paracetamol-induced acute liver injury *via* enhancing antioxidant levels and inhibiting lipid peroxidation.

Proinflammatory cytokines (IL-6, IL-1β, and TNF-α) and their abnormal metabolism play a significant role in the pathogenesis of ALD (47). Earlier, Khan et al. (48) reported that ethanol administration led to the activation of NF-κB and elevated the TNF-α levels in the liver. With the consistence of Khan et al. (48), we also observed the elevation of hepatic proinflammatory cytokines levels (IL-6, IL-1β, and TNF-α) in the M group, clearly indicating that inflammatory factors play a major role in ALD. Interestingly, ellagic acid administration (EL and EH) could reduce the inflammatory factors in the hepatic tissues of ALD mice. Similarly, intervention with bioactive compounds and natural products could also reduce the inflammatory responses associated with acute alcoholic liver injury (18–20, 32, 35, 48).

In this study, histopathological findings showed a significant alteration in the liver architecture, edema, hepatocytes degeneration, and inflammatory cells infiltration in ALD mice. Interestingly, the administration of ellagic acid markedly improved histopathological features of ALD mice. These results were consistent with the previous reports of bioactive compounds protecting the liver from the toxic effects of alcohol (18–20, 43, 44, 49).

It has been reported that TLR4 plays a critical role in the development and pathogenesis of ALD *via* inducing inflammatory cytokine, TLR adapter, and the expression of MyD88 (50, 51). TLR4 has the potential to activate two different pathways, such as MyD88-independent/TRIF-dependent and MyD88-dependent pathways (52). Our results revealed that alcohol administration activated the TLR4 pathway *via* upregulating hepatic MyD88 and TLR4 genes expression. However, ellagic acid supplementation markedly decreased the hepatic genes expression of MyD88 and TLR4. Earlier, Lee et al. (53) demonstrated that ellagic acid could ameliorate concanavalin A-induced hepatitis through TLR4/MyD88/NF-κB signaling pathway. In another study, ellagic acid has also been reported to improve anxiety and sleep by inhibiting the TLR4 signaling pathway (54). Liu et al. (55) also reported that dioscin alleviated ALD in the hepatic stellate cell *via* the TLR4/MyD88/NF-κB signaling pathway. Similarly, many bioactive compounds were also reported to reduce the inflammatory responses *via* TLR4/NF-κB-mediated inflammatory pathway (56).

As stated in the introduction, alcohol consumption can elevate bacterial endotoxin levels, which disrupts intestinal integrity and barrier function, and significantly promotes the occurrence of ALD. In addition, alcohol consumption also changes the gut microbiota composition, leading to abnormalities in the gut–liver axis (6, 8, 57). Moreover, it has been documented that some intestinal microbiomes, such as *Bacteroides* and *Akkermansi*a, have beneficial properties and can regulate inflammation (13, 58). Our results showed significant microbiota dysbiosis following ethanol exposure, which were consistent with previous studies (43, 44, 49, 59). As previously described in results sections, the relative abundance of microbiota such as *Firmicutes, Verrucomicrobia, Actinobacteria, Bacteroidetes*, and *Proteobacteria* at phylum level and *norank_f__Muribaculaceae, Lactobacillus, Kurthia, Akkermansia, Bacteroides*, and *Lachnospiraceae_NK4A136_*group at genus level were significantly altered after the intake of alcohol, which were further restored after ellagic acid treatment. Previously, it was reported that *Lactobacillus* species play an important role in the pathogenesis of ALD. The long-term alcohol intake decreases the *Lactobacillus* species abundance *via* altering the FFA level in serum circulation (49), which is relevant to our findings. Moreover, *Lactobacillus* species were also reported to exclude pathogens, stimulate mucin secretion, and regulate inflammatory responses (60). The ellagic acid supplementation increased the *Lactobacillus* species abundance. Okra seed oil supplementation could increase the *Lactobacillus* species population in ethanol-induced ALD mice (44). Similarly, lychee (*Litchi chinensis Sonn.)* pulp phenolic extract was also reported to modulate *Lactobacillus* species in ALD mice (43). The consumption of phenolic compounds (e.g., *p*-coumaric acid, caffeic acid, sinapic acid, rutin, hesperidin, and ferulic acid) and food rich with bioactive compounds (rhubarb, okra seed oil, and lychee) contributed to the increment of healthy gut microbiota in animal experiments (43, 44, 49, 59, 61–66). It is well-known that phenolic compounds are metabolized *via* microbiota in the colon but are not absorbed directly, thus contributing to the balance of gut microbiota (63, 64). Together, all these data suggest that ellagic acid could modulate the alcohol-induced gut microbiota dysbiosis.

In the present study, we demonstrated that ellagic acid (low and high doses) can attenuate alcohol-induced liver injury in mice. The ellagic acid supplementation exerted hepatoprotective effects *via* improving oxidative stress, decreasing inflammatory responses, and modulating gut microbiota composition. According to the above results, we can conclude that ellagic acid has strong potentials against ALD. Our results divulged that ellagic acid may be an ideal nutraceutical ingredient to prevent ALD. However, further mechanistic studies should be carried out prior to its clinical application.

## Data Availability Statement

The datasets presented in this study can be found in online repositories. The names of the repository/repositories and accession number(s) can be found below: NCBI Sequence Read Archive (SRA) under the BioProject number PRJNA752701. The website is https://www.ncbi.nlm.nih.gov/bioproject/?term=PRJNA752701.

## Ethics Statement

The animal study was reviewed and approved by Ethics Committee of the Beijing Key Laboratory of Functional Food from Plant Resources.

## Author Contributions

LZ, AM, and CW had contributed to data collection and analysis, and manuscript preparation. AI, MI, SA, and MS supervised the whole study and revised the manuscript. All authors contributed to the article and approved the submitted version.

## Funding

This research was funded by the China Postdoctoral Science Foundation (2019TQ0011), Beijing Postdoctoral Research Foundation, Technological Innovation Service Capacity Building-Basic Scientific Research Expenses (PXM2020-014213-000017), and Taif University Researchers Supporting Project (TURSP-2020/105).

## Conflict of Interest

The authors declare that the research was conducted in the absence of any commercial or financial relationships that could be construed as a potential conflict of interest.

## Publisher's Note

All claims expressed in this article are solely those of the authors and do not necessarily represent those of their affiliated organizations, or those of the publisher, the editors and the reviewers. Any product that may be evaluated in this article, or claim that may be made by its manufacturer, is not guaranteed or endorsed by the publisher.
